# Emotional Voice and Emotional Body Postures Influence Each Other Independently of Visual Awareness

**DOI:** 10.1371/journal.pone.0025517

**Published:** 2011-10-07

**Authors:** Bernard M. C. Stienen, Akihiro Tanaka, Beatrice de Gelder

**Affiliations:** 1 Laboratory of Cognitive and Affective Neuroscience, Tilburg University, Tilburg, The Netherlands; 2 Waseda Institute for Advanced Study, Tokyo, Japan; 3 Martinos Center for Biomedical Imaging, Massachusetts General Hospital and Harvard Medical School, Charlestown, Massachusetts, United States of America; University of Reading, United Kingdom

## Abstract

Multisensory integration may occur independently of visual attention as previously shown with compound face-voice stimuli. We investigated in two experiments whether the perception of whole body expressions and the perception of voices influence each other when observers are not aware of seeing the bodily expression. In the first experiment participants categorized masked happy and angry bodily expressions while ignoring congruent or incongruent emotional voices. The onset between target and mask varied from −50 to +133 ms. Results show that the congruency between the emotion in the voice and the bodily expressions influences audiovisual perception independently of the visibility of the stimuli. In the second experiment participants categorized the emotional voices combined with masked bodily expressions as fearful or happy. This experiment showed that bodily expressions presented outside visual awareness still influence prosody perception. Our experiments show that audiovisual integration between bodily expressions and affective prosody can take place outside and independent of visual awareness.

## Introduction

Our social interactions depend on receiving and combining affective signals from multiple sources such as faces, voices, body postures and other contextual information in our environment. Previous research has mainly investigated face-voice combinations [1], [2], [3], [4]. For example, de Gelder & Vroomen [3] presented facial expressions that were morphed on a continuum between happy and sad, while at the same time a short spoken sentence was presented. This sentence had a neutral meaning, but was spoken in either a happy or sad emotional tone of voice. Participants were instructed to attend to and categorize the face, and to ignore the voice, in a two-alternative forced-choice task. The results showed a clear influence of the task-irrelevant auditory modality on the target visual modality.

More recently body-voice combinations have also been studied [5], [6] generalizing these multisensory effects to a broader domain. Results from a number of behavioural experiments using independent stimulus sets now allow us to conclude that recognition performance for bodily expressions is very similar for face and body stimuli. By switching to a new affective stimulus category, we may be capable of extending the scope of face-based research and provide evidence that human emotion theories may generalize to other affective signals as well. A major difference between facial and bodily expressions is that the latter can be recognized from far away while the former require the viewer to be nearby. This is potentially an important difference between how facial and bodily expressions play their communicative roles and it should have consequences how the specific information is conveyed [7], [8].

Crossmodal emotion effects are shown whereby affective information in one sensory modality influences perception in the other while the signals are perceived both consciously [3], [9]. These crossmodal effects have again mainly been shown for faces. However, previous studies on the automaticity of audiovisual integration have mainly investigated the role of attention [10], [11]. But attentional selection does not imply that one is consciously aware of the stimulus. Also, the unattended stimulus could be consciously perceived [12]. This uncontrolled role of consciousness could explain why multisensory integration occurs. For example, if consciousness is necessary for multisensory integration to occur then the process is not automatic. There is some evidence that visual awareness does not seem to be a prerequisite for audiovisual affect integration since crossmodal interactions are still observed when the face is not consciously perceived in hemianopic patients [13], but, so far, if this is the case in neurological intact observers remains unknown.

A number of research reports have concluded that emotional information can be processed without observers being aware of it. Many studies using facial expressions now provide direct and indirect evidence for visual discriminations of affective stimuli in the absence of visual awareness of the stimulus. Clinically blind hemianopic patients have shown on forced choice tasks that they can reliably guess the emotion not only of facial but also of bodily expressions presented in their blind field [14], [15].

Masking is one of the most widely used techniques for exploring unconscious processing of visual emotional information in neurologically intact observers. For example, Esteves and Öhman [16] found that short (e.g. 33 ms) presentation of a facial expression (happy and angry) replaced immediately by a neutral face (mask) with a longer duration (e.g. 50 ms) is below the participants' identification threshold. We have recently shown in a parametric masking study that the detection of fearful bodily expressions covaries less with visual awareness than the detection of other bodily expressions [17].

Öhman [18], [19] suggests that fear stimuli automatically activate fear responses and captures the attention as shown in visual search tasks where participants had to detect spiders, snakes or faces among neutral distracters [20], [21]. The special status of fear stimuli is still a matter of debate, specifically in relation to the role of the amygdale [22], [23].

Here our goal was to address whether affective information from voices influences the affective information from bodily expression independently of visual awareness. First, we investigated the influence of the perception of emotional voices on the recognition performance of emotional body expressions under conditions of visual uncertainty, and subsequently we investigated whether unseen bodily expressions affect the recognition of the prosody in the perceived voice.

## Results and Discussion

### Experiment 1: emotional voice influences bodily expression categorization independently of visual awareness

**Figure 1:**
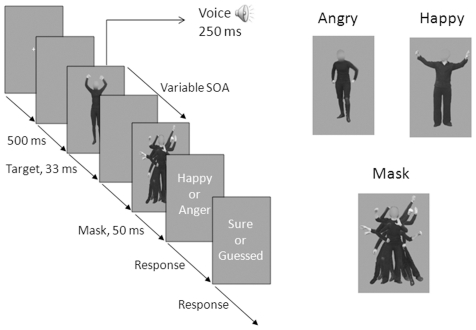
Illustration of an example trial and example stimuli (experiment 1). An example trial (*left*), an example of an angry and happy bodily posture (*upper right*), the mask (*below right*).

In this experiment a mask was presented at 12 different latencies after or before the onset of the target (Stimulus Onset Asynchrony, SOA), which were angry or happy bodily expressions. The participants were instructed to categorize bodily expressions which were congruently or incongruently paired with emotional voices and subsequently to indicate whether they were sure of their answer or whether they were guessing. Importantly, instructions specified they had to ignore the voice. See Figure 1 for a schematic representation of a trial and for examples of the stimuli.

Percentage correct categorized bodily expressions were corrected for chance level which was 50 percent. To assess whether participants could differentiate between the correct and incorrect answers confidence ratings were calculated. The number of sure responses when the categorization of the emotional expression was incorrect was subtracted from the number of sure responses when the response was correct. This was divided by the total number of correct and incorrect answers. A resulting value of zero would mean that the participants indicate subjectively that they are not more confident of their correct answers then their incorrect answers which is taken as a measure of subjective visual awareness. A similar approach was chosen by Cheesman and Merikle [24] and Esteves and Öhman [16] as a measure of the phenomenological experience of the participants' perception of the targets.

This method automatically controls for how well the participants are engaged in the task. If, for example, a participant would just randomly categorize the emotion, but always indicates to be sure, the confidence measure would end up being 100 percent while the accuracy would be around zero after correction of chance level. However, our measure of confidence would also result in a confidence rating of zero, because it automatically corrects for when the participants indicate to be sure, while their answer is wrong.

Two participants were discarded from analysis because they performed well below 50 percent in categorizing the angry and happy bodily expression in the validation study (37.5 and 25.0 percent), while the group average was 84.4 percent (SD = 13.9 percent). The correct identifications were on such a low level that there is a possibility that the two participants did not understand the instruction clearly, for example they confused the order of the response buttons. The validation study showed that the angry bodily emotion was correctly identified 84.8 percent of the cases (SD = 16.3) and happy bodily emotion 83.9 percent of the cases (SD = 19.3).

**Figure 2:**
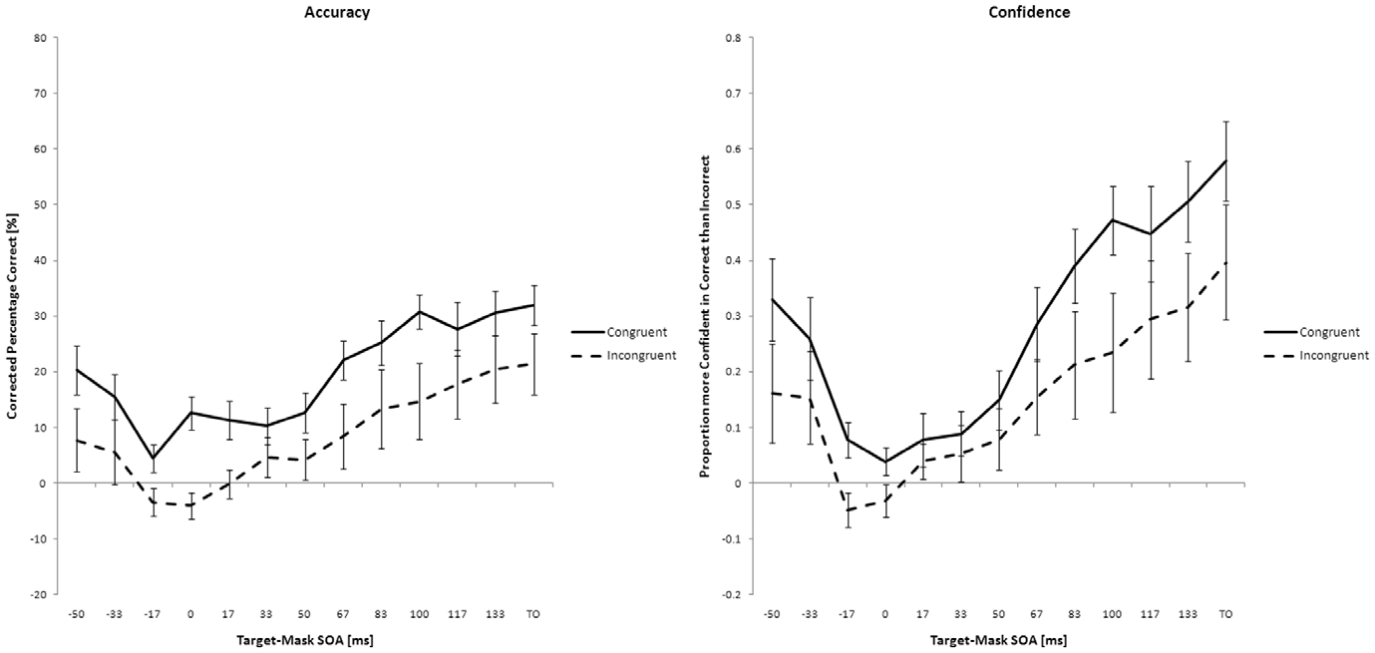
Results of experiment 1. *Left*: Mean categorization performance plotted as function of SOA latency corrected for chance (50 percent). *Right*: Mean confidence ratings plotted as function of SOA latency. Error bars represent standard error of the mean. SOA = Stimulus Onset Asynchrony, TO = Target Only.

Two GLM repeated measures analyses with emotion (2 levels), congruency (2 levels) and SOA latency (13 levels) as factors were performed on the categorization performance and confidence ratings. There was a main effect of SOA latency and congruency on accuracy, resp. F(12,156) = 14.50, p<.01; F(1,13) = 10.45, p<.01. Also, a main effect of SOA latency and congruency were observed on the confidence ratings, resp. F(12,156) = 18.67, p<.01; F(1,13) = 10.96, p<.01. Bonferroni corrected pairwise comparisons showed that the longer the SOA latency the higher the categorization performance and confidence ratings, e.g., when there was no mask in the case of the target-only trials the categorization performance was highest (mean = 76.7%, SD = 4.02) and lowest when the SOA latency was −17 ms (mean = 50.5%, SD = 1.20). For the confidence ratings this was also true. The participants were most confident when there was no mask (mean = .557, SD = .083), although they were the least confident when the SOA latency was 0 ms (mean = .019, SD = .019). In addition the comparisons between incongruent and congruent body-voice pairs showed that the categorization performance and confidence ratings were higher when the emotion was congruent. The specific emotion did not have a main effect on the accuracy or confidence ratings nor did it interact with the other factors. Figure 2 shows the accuracy and the confidence of the participants averaged over the two emotions.

Interestingly, there was no interaction between congruency and SOA latency on accuracy (*F*(12, 156) = 1.09, *p* = .37), while the factors interacted on confidence ratings (*F*(12, 156) = 2.48, *p*<.01). To investigate this interaction post hoc comparisons were done between congruent and incongruent trials on the confidence ratings per SOA latency. Results suggested that the difference between congruent and incongruent trials was absent in the confidence ratings when the SOA latency ranged from 0 to +50 ms (*p*>.05, Bonferroni corrected). Within this range it appeared that when the SOA latency ranged from 0 to +33 ms the confidence ratings when the emotion of voice and body were congruent or incongruent were never above zero (all *p*>.0125, Bonferroni corrected). Yet, when the emotion of the voice-body pairs was congruent the accuracy in the whole range (from 0 to +50 ms) was above zero (all *p*<.0125, Bonferroni corrected), while this was not the case when the emotion of the voice-body pairs were not congruent (all *p*>.0125, Bonferroni corrected).

The results show that when emotional voices and body postures are congruent objective recognition of emotional body expressions is aided regardless of SOA latency. This same effect is not seen in subjective confidence ratings where there is no facilitation effect of congruent voice information for short SOA latencies. Conjointly, the confidence of the participants was not above zero in this range while the accuracy when the emotional voice-body pairs were congruent was above chance. The subjective ratings can be taken as measure of the phenomenological experience of the participants' perception of the targets [16], [24]. The combination of these findings shows that the emotion of the voice exerts its influence independently of the visual awareness of the target.

Also, the lack of the interaction between congruency and SOA latency in accuracy shows that these results do not reflect merely a decision or response bias [1]. Such a bias would be stronger when visibility of the target is low and would thus result in an interaction of congruency and SOA latency on the categorization performance of the participants. In other words, this method shows to be a very good control to check whether such a bias is present in the data set.

While this study shows that visual awareness is not necessary for the multisensory integration to occur the participants were in fact, capable of detecting the bodily expressions in the majority of the trials because this concerns a parametric masking study. In other words, they were aware that bodily expressions were presented while ignoring the human emotional vocalizations. In a second study we therefore isolated one SOA condition in order to ensure that the participants would not perceive bodily expressions throughout the whole experiment while judging the emotion of spoken sentences. If we would observe similar effects on the judgment of emotional prosody because of the influence of unseen bodily expressions this would strengthen the conclusion that bodily expressions and emotional voices influence each other independently of visual awareness.

### Experiment 2: unseen bodily expressions influence interpretation of emotional prosody in the voice

**Figure 3:**
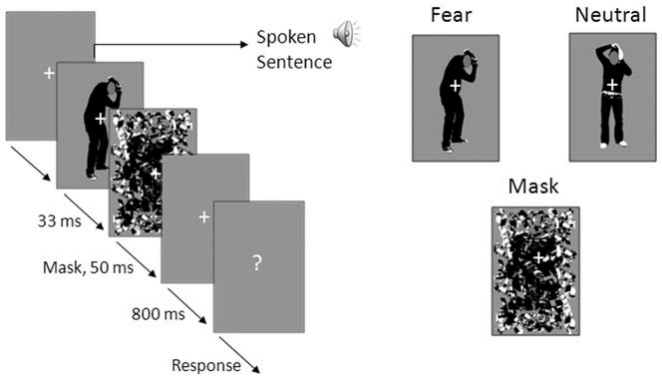
Illustration of an example trial and example stimuli (experiment 2). An example of a trial of experiment 2 (*left*), an example of a fearful bodily expression and a neutral action (*upper right*) and the mask (*below right*).

In the first experiment the influence of the emotion in the voice and its dependency on visual awareness was the focus of interest. In this second experiment we asked whether bodily expressions when presented outside visual awareness can influence the recognition of prosody in spoken words. While in the first experiment the visibility of the bodily expressions was parametrically varied we held the SOA latency constant (33 ms) in this experiment. Participants had to focus on the voice component of the stimulus which consisted of different levels of emotion on a 7-step continuum between fearful and happy. They were instructed to categorize the emotion in the voice clip. Visual catch trials were introduced to make sure that the participants were looking at the computer screen where masked bodily expressions were presented. Our extensive semi-structured exit interview and our sensitive post test assessed whether the participants had been aware of the emotional body pictures. See Figure 3 for a schematic representation of a trial and examples of the stimuli.

Seven out of thirty-two participants were excluded from analysis because their score was higher than .11 on the post test. See the method section how the score was begin threshold. These participants also indicated in the exit interview having seen several body stimuli.

One participant was discarded from analysis because he missed 26 percent of the catch trials (group mean = 2.0%, SD = 4.7%). In the validation session the fearful bodily expressions were correctly identified in 92.7 percent of the cases (SD = 15.6) and the neutral action was correctly identified in 95.8 percent (SD = 12.0) of the cases.

**Figure 4:**
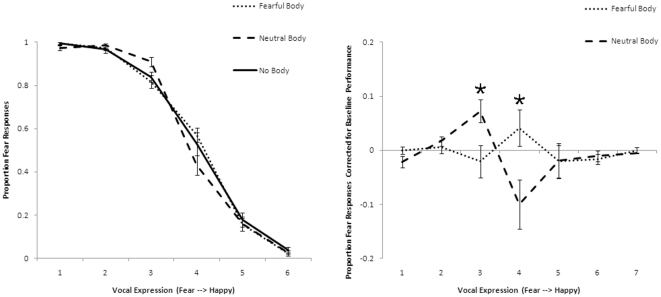
Results of experiment 2. *Left:* Fear responses as a function of morphed emotional spoken sentences when masked neutral actions, fearful bodily expressions or no bodies were shown. *Right:* Fear responses corrected for baseline performance (no-body trials) as a function of morphed emotional spoken sentences when masked neutral actions or masked fearful bodily expressions were shown. Error bars represent standard error of the mean. Asterisks indicate *p*<.001.

The no-body masked condition was used as baseline. The number of fear responses were corrected for this baseline performance per morphed emotional voice condition separately for masked fearful bodily expressions and neutral bodily actions, see Figure 4. A value of zero meant that the emotional sentence was not more or less categorized as fearful when a fearful bodily expression or a neutral action was shown in comparison to when no masked bodily stimulus was presented. A 2 (fearful bodily expression, neutral action) * 7 (voice clips) GLM repeated measures analysis indicated a significant interaction between the masked bodily expressions and the voice clips on the fear responses (*F*(3,61) = 8.11, *p*<.001, the Greenhouse–Geisser epsilon is reported because sphericity could not be assumed). This shows that the masked body stimuli influenced the categorization of the emotion in the voice and that this difference depended on which morphed sentence was presented to the participants. Bonferroni corrected paired t-testing (7 comparisons, thus α = .05/7 = .007) were performed between fear responses to the voice when fearful or neutral bodily expressions were presented. This revealed that when the voice was slightly more fearful than happy and masked neutral pictures were presented participants categorized the voice more as being fearful (mean = .07, SD = .14) than when masked fearful pictures were presented (mean = −.021, SD = .16), t(23) = −3.252, *p* = .004. Interestingly, when the voice was a 50/50 morph between fearful and happy participants classified the voice more as being fearful when masked fearful bodily expressions were presented (mean = .04, SD = .25) in comparison to when masked neutral bodily actions were presented (mean = −.10, SD = .23), t(23) = 3.129, *p* = .005. See Figure 4.

We were primarily interested whether the bodily expression while unseen exerts its influence on the perceived emotion in the voice. Importantly, this study revealed that when fearful bodily expressions and neutral actions are presented outside visual awareness they still influence the interpretation of the prosody in spoken words. Unseen fearful bodies triggered more fear responses when the emotion of the spoken sentence was a 50/50 morph of both emotions.

The results leave us wondering why fear responses increased when the voice was slightly more fearful but the unseen bodily expression was neutral. It may be the case that this is caused by the mismatching of the emotional dimensions of the two sensory signals. The ambiguity that is introduced when the voice is fearful but the visual stimulus is neutral could have confused the participants. The unseen neutral bodily expressions did not deliver extra information which could help processing the auditory signal. Alternatively, it might be that although the validation results were very good, on an unconscious level the neutral bodily expression might be perceived as being fearful. This is a possibility which suggest further research on this intriguing question like developing a stimulus set which is not only validated explicitly but also with the use of autonomous responses such as pupil dilation or skin conductance.

The duration of the vocal stimulus was much longer than the duration of the masked visual stimulus. Although this study mainly focused on the influence of masked bodily expressions on the processing of overtly presented verbal sentences the large discrepancy might have attenuated the effect skewing the results towards the vocal stimuli. It might be that with shorter clips such as were used in experiment 1 lead to larger effects.

### General Discussion

Our goal was to investigate whether the emotional voice influences the recognition of emotional bodily postures independently of visual awareness and whether unseen emotional bodily expressions influence the recognition of the emotion expressed in the voice. The results of the first experiment showed that a dissociation occurred between objective and subjective measures. When SOA latencies were short the objective categorization performance was still facilitated by the congruent emotional voice while this facilitation effect was absent in the subjective confidence ratings. We conclude that the emotional voice influences the categorization of emotional body postures independently of visual awareness because participants seemed not to be aware while they were categorizing the emotional bodies above chance. The second experiment showed that bodily postures presented outside visual awareness still influenced the interpretation of the emotion in the voice. When the bodily expression was fearful participants categorized the voice as being more fearful when the voice was a 50/50 morph between fearful and happy. Surprisingly masked neutral bodily actions triggered more fear responses to the voice than when the voice was already slightly more fearful.

In the second experiment a trial-by-trial measurement would have been possible except that this conflicts with the goal to present bodily expression outside the visual awareness of the participants. Therefore we combined an extensive semi-structured exit-interview with a sensitive post test. During the exit-interview it was ensured to give the participants as much space as possible to express their experience they had during the experiment. If there was just the smallest hint towards reporting any bodily postures or even objects, it resulted in exclusion from analysis. In addition, we applied a strict criterion to the post test which dictated that if any of the emotional postures was reported as seen, the participant was excluded from the analysis. Given the fact that only 7 out of 31 participants were excluded while the criteria were strict and the tests were sensitive it supports our assertion that the masking of the targets was effective.

Our findings are consistent with earlier studies showing the crossmodal influence of human emotional sounds on the recognition of emotional body postures [6] and the influence of emotional body postures on the interpretation of voice prosody [25]. The study performed here adds the important notion that this crossmodal interaction is even taking place when the observer is not aware of the visual information. In addition, emotional information from one modality can influence the emotional information from another modality independently of visual awareness.

The influence of facial expressions of which there is no sensory awareness on the processing of emotional voices was already shown in hemianopic patients [13]. Our study now generalizes these findings to healthy participants and to bodily expressions. When conscious processing of visual signals by the cortical mechanisms via the striate cortex is prevented, the colliculo-thalamo-amygdala pathway could still process the stimulus. This was already shown in recent fMRI studies that have suggested differential amygdala responses to fearful faces as compared to neutral faces when the participants were not aware of the faces [26], [27]. It would be interesting to evaluate these processing pathways in the light of the current study to shed light on the neurofunctional basis of how these signals interact in absence of visual awareness.

Also, future research should reveal how the results of the present study generalize to other emotions and different contexts to investigate the influence of environment on the affective multisensory integration. In addition, it would be interesting to see how the integration of other sensory modals is influenced such as haptics or smell. This field of research will give rise to insights in that affective signals often require a rapid reaction from the observer and intersensory redundancy, so it is assumed, contributes to speed by reducing uncertainty.

## Methods

### Experiment 1

#### Participants

Sixteen undergraduate students of The University of Tilburg participated in exchange for course credits or a monetary reward (9 women, 7 men, M = 20.0 years, SD = 2.2). All participants had normal or corrected-to-normal vision and gave informed consent according to the declaration of Helsinki. The protocol was approved by the local Ethics Committee Faculteit Sociale Wetenschappen of Tilburg University.

#### Stimuli and procedure

Frames from video clips were used as stills of bodies displaying angry and happy expressions. For full description of the set of video clips and information regarding their validation see [28]. In total 16 stimuli (2 emotions×2 gender×4 actors) were selected. These stimuli were frames from the video clips in which the actor seemed to be optimally expressing the emotion. The faces of the actors were covered to prevent that the facial expression would influence the identification of the emotional body posture. All actors wore black clothing and all images were converted to grey values.

Still images taken from neutral action video clips such as fixing one's hair or cloths were selected to construct the mask. A neutral bodily expression of a male with an average posture was chosen as the basis. The arms and legs were erased and twelve arms and legs from other identities expressing a neutral emotion were attached to the body at different positions and orientations creating the image of a body with more arms and legs than usual.

Average height of the bodies was 7.82 degrees (SD = .26 degrees), the average maximum width (distance between the hands) was 3.76 degrees (SD = .85 degrees) and the average waist was 1.55 degrees (SD = .14 degrees) of visual angle. The height of the mask was 8.12 degrees, the maximum width was 6.21 degrees and the waist was 1.64 degrees of visual angle. The mask covered the target stimuli completely. See Figure 1 for examples of the stimuli.

Twenty-four emotional meaningless human vocalizations (e.g., “ah” or “uh”) expressing happy or angry emotions from 12 different speakers were recorded. Each recording was edited to create 8 different fragments of 8 different durations (25, 50, 75, 100, 150, 200, 250, and 400 ms), resulting in 192 stimuli in total. Loudness was equated in terms of the A-weighted sound pressure level. Sounds were gated with 5 ms raised-cosine onset and offset ramps in order to avoid clipping. In the pilot experiment, 10 participants categorized the emotion of all the 192 vocal expressions into happy or angry emotions. Based on the accuracy results, we decided to use the voice clip of 250 ms for which the overall accuracy was highly above chance (89.8%), t(9) = 15.23, *p*<.001.. The accuracy results did not differ between emotions, t(3) = 0.77, p = .50. Angry and happy vocalizations from two male and two female speakers (e.g., “ah” or “uh”) were used and paired congruently and incongruently with the visual stimuli. The voice-body stimulus compound was always gender-congruent.

Participants were comfortably seated in a chair in a soundproof experimental booth approximately 90 cm from the screen. The disappearance of a fixation cross signaled the beginning of a trial. After 500 ms the target stimulus appeared for 33 ms accompanied with an angry or happy voice, which was congruent or incongruent (50 percent/50 percent) with the emotion of the bodily posture. After a variable interval the mask was presented for 50 ms (in case of forward masking the mask was presented first).

It is known that the largest masking magnitude associated with pattern masking is around an SOA latency of 0 milliseconds [29], [30]. Therefore the values for the SOA latencies included the SOA latency of 0 ms. The SOA latencies were −50, −33, −17, 0, 17, 33, 50, 67, 83, 100, 117 and 133 ms. Negative values represent forward masking and positive values backward masking. When the SOA latency was −33, −17, 0 and17 ms the target overlaps with the mask. The target was always presented at the foreground. Moreover a target-only condition and a no-target condition were included.

The participants were instructed to categorize the emotional expressions of the body and to ignore the emotional voice. They had to respond with the left hand using two response buttons situated in front of them with the labels “Happy” and “Angry” attached to it. Subsequently they had to indicate whether they were sure or guessing. They had to respond with the right hand with two different buttons on the same response box labeled with “Sure” and “Guessed”. They were instructed to use their “gut feeling” if they had not seen the body. Fingers, but not hands were counterbalanced across participants. See Figure 1 for a schematic representation of a trial.

Previous to the experimental sessions the participants performed three practice sessions consisting of 27 trials each. Other identities than the ones used in the main experiment served as targets. When the participants did not miss trials and gave notice of a full understanding of the procedure the main experiment was started. A total of four runs were presented adding up to a total of 896 trials. Every 112 trials there was a 3 minute break. After the main experiment in a separate session all targets were presented for 33 ms without the pattern mask in order to validate the stimuli. The participants were instructed to categorize the targets in angry and happy expressions. The total duration of the experiment was 1 hour and 45 minutes.

### Experiment 2

#### Participants

Thirty-two undergraduate students of Tilburg University participated in exchange of course credits or a monetary reward (20 women, 11 men, M = 20.4 years, SD = 1.8). All participants had normal or corrected-to-normal vision and gave informed consent according to the declaration of Helsinki. The protocol was approved by the local Ethics Committee Faculteit Sociale Wetenschappen of Tilburg University.

#### Stimuli and procedure

Eight photos of four male actors expressing fear or combing their hair were selected from a well validated photoset described in [17]. The stimuli were from the same set as described in experiment 1 with the exception that the colors were saturated to white and black. This was done to remove extra line elements because of the wrinkling of the clothing of the actors making it easier to mask the bodily expressions. Average height of the bodies was 8.14 degrees (SD = .35 degrees), the average maximum width (distance between the hands) was 3.12 (SD = .25 degrees) degrees and the average waist was 1.57 degrees (SD = .07 degrees) of visual angle. See Figure 3 for examples of the stimuli.

The auditory stimuli consisted of a Dutch spoken sentence “met het vliegtuig” (which means “with the plane”), edited so as to express different levels of emotion on a 7-step continuum between fearful and happy. The editing consisted of adjusting the duration, pitch range and pitch register. The voice clips lasted on average 792 ms (SD = 51 ms). See for more details [3].

Thus, the emotional dimension was only matched for fear and not for happy. The main reason was that we conjectured that if the unseen bodily expressions were both emotional this could lead eventually to a mixed effect. If in one trial the emotional expression would be happy and in the other it would be fearful the effect on the participants would be unpredictable. When only using neutral and fearful bodily expressions one can be sure that if there would be an effect, it would be in the direction of fear induction.

A pattern mask was constructed by cutting the target bodies into asymmetric forms which were scrambled and replaced in the area occupied by the bodies (see Figure 3). The rationale behind creating a new mask for this study was to avoid inducing any percept of a body. The mask measured 9.85 by 6.48 degrees of visual angle and completely covered the area of the stimuli.

A trial started with a white fixation cross on a gray background. After 500 ms a voice clip was presented. On the onset of this voice clip the masked fearful bodily expression, the neutral bodily action or the no-body (mask) stimulus was presented for 33 ms and subsequently the mask for 50 ms. The no-body condition was added to create a baseline in which neither the neutral action or the fearful expression was presented, instead the mask was presented for 88 ms. In 22 percent of the trials the fixation cross turned 45 degrees clockwise and switched back to the original position after 133 ms. See Figure 3 for a schematic example of a trial.

The participants were instructed to categorize the emotion in the voice clip as fearful or happy. Whenever the cross turned clockwise they had to withhold their response. This functioned as a catch trial to make sure that the participants were looking at the screen when the displays of emotional body postures were presented. The participants were told that we were interested whether the recognition of emotion in the voice is influenced when the perceptual system is loaded with visual information. This was done in order to provide the participants with a reasonable explanation why they saw the mask during the experiment and why the catch trials were presented as well as it ensured that they were naive to the actual goal of the experiment.

There were two experimental runs with a total of 216 trials (2 runs consisting of 108 trials: 4 identities×3 masking conditions (fearful expression, neutral action, no-body)×7 (emotional voice)+24 catch trials). Every 54 trials there was a 2 minute break. The experiment was preceded with a practice session and was followed by a short validation session. The total duration of the experiment was 1 hour.

In order to check whether the participants had been unaware of the body stimuli we conducted an extensive semi structured exit interview and a sensitive post test. In the exit interview we began by asking general questions such as “What do you think about the experiment?” and subsequently tuned in to find out whether the participants had been aware of the body stimuli. The questions ranged from “Have you noticed anything during the experiment?” to “Have you been distracted by anything?” to finally just asking them “Have you seen for example footballs, faces, bodies or shoes?”. Only participants that never indicated having seen a body stimulus or even something like an object were included in the analysis.

Finally, in a post test the 9 stimuli that were used in the main experiment (4 male fearful expressions, 4 male hair combing actions and the mask) and 40 new bodily expressions (4 female fearful expressions, 4 male and female angry bodily expressions, 4 male and female happy bodily expressions, 4 female hair combing actions, 4 male and female phoning actions and 4 male and female drinking actions) were presented. The participants were instructed to classify the stimuli as seen if they recollect that they have seen the bodily posture during the main experiment and as not seen when they could not recollect the bodily posture. The stimuli all were presented twice and the presentation duration was 33 ms which was enough to clearly see the body. Proportion classified as seen when it was a new stimulus was subtracted from proportion classified as seen when it was an old stimulus. Because the masks were included in the post test and it was possible to detect the masks very easily during the main experiment it was expected that the participants would at least identify the masks. This would result in having seen 2 out of totally 18 of the stimuli used in the experiment and 0 out of totally 80 of the new bodily expressions. The resulting value would then be .11 (2/18–0/80). Participants scoring above .11 were excluded from the analysis.
